# Creatinine-or cystatin C-based equations to estimate glomerular filtration in the general population: impact on the epidemiology of chronic kidney disease

**DOI:** 10.1186/1471-2369-14-57

**Published:** 2013-03-12

**Authors:** Pierre Delanaye, Etienne Cavalier, Olivier Moranne, Laurence Lutteri, Jean-Marie Krzesinski, Olivier Bruyère

**Affiliations:** 1 Department of Nephrology-Dialysis-Transplantation, University of Liège, CHU Sart Tilman, Liège 4000, Belgium; 2 Department of Clinical Chemistry, University of Liège, CHU Sart Tilman, Liège, Belgium; 3 Division of Nephrology and Public Health, CHU de Nice, Nice, France; 4 Department of Public Health, Epidemiology and Health Economics, University of Liège, CHU Sart Tilman, Liège, Belgium

**Keywords:** Creatinine, Cystatin C, Chronic kidney disease, Glomerular filtration rate

## Abstract

**Background:**

Chronic kidney disease (CKD) is a major issue in public health. Its prevalence has been calculated using estimation of glomerular filtration rate (GFR) by the creatinine-based equations developed in the Modified Diet in Renal Disease (MDRD) and Chronic Kidney Disease Epidemiology Collaboration (CKD-EPI) study. Recently, new equations based either on cystatin C (CKD-EPI Cys) or both cystatin and creatinine (CKD-EPI mix) have been proposed by the CKD-EPI consortium. The aim of this study was to measure the difference in the prevalence of stage 3 CKD, defined as an estimated GFR less than 60 mL/min/1.73 m^2^, in a population using these four equations.

**Methods:**

CKD screening was performed in the Province of Liège, Belgium. On a voluntary basis, people aged over 50 years have been screened. GFR was estimated by the four equations. Stage 3 CKD was defined as a GFR less than 60 mL/min/1.73 m^2^.

**Results:**

The population screened consisted of 4189 people (47% were men, mean age 63 ± 7y). Their mean serum creatinine and plasma cystatin C levels were 0.88 ± 0.21 mg/dL and 0.85 ± 0.17 mg/L, respectively. The prevalence of CKD in this population using the MDRD, the CKD-EPI, the CKD-EPI Cys and the CKD-EPI mix equations was 13%, 9.8%, 4.7% and 5%, respectively. The prevalence of CKD was significantly higher with the creatinine-based (MDRD and the CKD-EPI) equations compared to the new cystatin C-based equations.

**Conclusions:**

Prevalence of CKD varies strongly depending on the method used to estimate GFR. Such discrepancies are of importance and must be confirmed and explained by additional studies, notably by studies using GFR measured with a reference method.

**Trial registration:**

B70720071509

## Background

Chronic kidney disease (CKD) is frequently presented as a major public health issue, because a prevalence as high as 10% has been described in the general population of Western Countries [1,2]. In addition, CKD is associated with a high mortality risk [3-5]. In this context, prevention and screening of CKD could be of importance, even if definitive proof of the benefits of such CKD screening is still lacking [6-8]. Disease screening requires the use of a diagnostic tool with high sensitivity and specificity. Measuring both glomerular filtration rate (GFR) and proteinuria are key elements to estimate the global function of the kidneys [9]. In this article, we will focus on the determination of GFR. Because measuring GFR may be costly and cumbersome, several authors have proposed creatinine-based equations to improve GFR estimation. The Modified Diet in Renal Disease (MDRD) study and the Chronic Kidney Disease Epidemiology Collaboration study (CKD-EPI) equations are used to estimate CKD prevalence in epidemiological studies [10,11]. For example, according to the National Health and Nutrition Examination Survey (NHANES) performed in the USA, the prevalence of stage 3 CKD (defined as an estimated GFR (eGFR) less than 60 mL/min/1.73 m^2^) was 6.3% and 7.8% using either the CKD-EPI or MDRD study equation, respectively [1,11]. However, there are several limitations to the use of serum creatinine-based equations. There are reasons to believe that both equations overestimate the real prevalence of CKD because they underestimate the measured GFR (mGFR) [12].

Plasma cystatin C is a relatively new marker of GFR. Cystatin C seems to have most of the characteristics of a good biomarker of GFR [13-15]. Several equations based on plasma cystatin C level have been created to estimate GFR [16]. However, these equations have been developed by the use of a limited sample of test subjects and have been poorly validated [13,15]. There is also concern because of the lack of standardized calibration for the measurement of the plasma level of cystatin C [17-21]. Now, a standardized calibration is available [22] and the CKD-EPI consortium has recently proposed equations (based on cystatin C alone or combined with creatinine) to better estimate GFR [23]. It is thus now possible to compare the prevalence of stage 3 CKD with the MDRD and CKD-EPI study equations to its prevalence based on the new cystatin C-based equations, namely the CKD-EPI based on cystatin C alone (CKD-EPI Cys) and CKD-EPI based on creatinine and cystatin C (CKD-EPI mix).

## Methods

This study was approved by the Ethics Committee of our University Hospital (Comité d’Ethique Hospitalo-Facultaire Universitaire de Liège). The Belgian registration number is B70720071509*.* This prospective study was performed in the context of the CKD screening program organized by the Department of Health of the Province of Liège. This province is one of the ten provinces in Belgium. Its area is 3862 km^2^ and its population in 2005 was 1,037,161 inhabitants. The CKD screening is enabled by a medical bus that travels through the 84 communes of the province. Between June 2008 and March 2010, 112,000 subjects without any other restriction that being aged between 40 and 75 years were invited through a personal letter to participate in a CKD screening. All individuals wanting to voluntary participate had to register and make an appointment by giving a phone call to the Department of Health of the Province of Liège. The time slot to visit the screening bus was from 9 am to 7.45 pm so that active working people could participate. We arbitrary limited our analysis to subjects older than 50 years. All patients have signed informed consent. Blood samples and anthropometric data were collected and a short interview was done. Between June 2008 and March 2010, frozen blood samples were sent to the Clinical Chemistry laboratory of the University of Liège where they were immediately thawed and assayed. Serum creatinine was measured by the IDMS traceable compensated Jaffe method (Roche Diagnostics, Mannheim, Germany) on Modular apparatus. Cystatin C was measured by a particle-enhanced nephelometric immunoassay (PENIA) on the BNII nephelometer (Siemens Healthcare Diagnostics, Marburg, Germany). The assay was not calibrated against the international certified reference material ERM-DA471/IFCC for cystatin C but values were multiplied by 1.11 as recommended by the manufacturer (customer notification November 18, 2010) to convert them to ERM-DA471/IFCC–equivalent concentrations [22,24]. Our university laboratory is currently accredited for the ISO 15189 Standard.

Diabetes mellitus was defined as glycosylated hemoglobin greater than 48 mmoL/moL or self-reported diabetes. Hypertension was defined as measured systolic blood pressure by oscillometric method of 140 mm Hg or greater, diastolic blood pressure of 90 mm Hg or greater or self-reported hypertension. Smoking status was self-reported.

The four equations analyzed in the study are shown in (Additional file 1: Table S1). Stage 3 CKD was defined as eGFR less than 60 mL/min/1.73 m^2^[9].

The data were anonymously analyzed. All results are expressed as mean ± SD. The percentage of CKD patients obtained with the four equations was compared with the McNemar test (paired samples). Agreement or reliability between GFR estimations was assessed by Cohen’s kappa statistics to discriminate GFR greater and less than 60 mL/min/1.73 m^2^ (only two possible categories) [25]. A kappa value of 0.20 or less was considered slight agreement; 0.21–0.40, fair agreement; 0.41–0.60, moderate agreement; 0.61–0.80, substantial agreement; and 0.81–1.00, almost perfect agreement. We compared the results of the four GFR estimations by paired sample *t*-test. The validity between eGFR was studied with estimation of the bias, SD (i.e. precision) and with a graphical approach (i.e. Bland et Altman) [26]. In these analyses, we have arbitrarily chosen the creatinine-based equation results as the reference value. Because no equation can be considered as the perfect reference, we have only considered the absolute bias (with neither positive nor negative sign). Bias between equations has been defined as the absolute mean of the differences. The SD around the mean reflect the dispersion of the equations.

Because performance of the equations and epidemiology of CKD are well known to be influenced by age, we performed analyses according to 10 years age bracket. We also repeated the analyses according to gender and diabetes status.

In subgroup analyses, we focused on discrepant results between the equations regarding the stage 3 CKD diagnosis (i.e. eGFR < 60 mL/min/1.73 m^2^). Clinical characteristics of discrepant subjects were compared by *t*-test and Chi square test. In all statistical data, we used p < 0.05 to determine statistical significance. All analyses were performed using MedCalc® (MedCalc Software, Mariakerke, Belgium).

## Results

During the study period, 4189 people were screened (47% male and 53% women). Anthropometric, clinical and biological characteristics of the population are shown in Table 1. By the paired samples *t*-test, four equations were all different from each other (p < 0.0001). Prevalence of different CKD stages is shown in Table 2. Prevalence of stage 3 CKD (defined as eGFR < 60 mL/min/1.73 m^2^) when GFR was estimated by the MDRD equation study was 13.1%. This prevalence was significantly higher than the prevalence obtained with the CKD-EPI (9.8%, p < 0.0001), the CKD Cys (4.7%, n = 198, p < 0.0001) and the CKD-EPI mix (5%, n = 210, p < 0.0001) equations. Differences between the CKD-EPI equations on one hand and the two other cystatin C-based equations on the other hand were also very significant (p < 0.0001). On the contrary, no significant difference was found between the CKD-EPI Cys and the CKD-EPI mix equations.

**Table 1:** Anthropometrical and biological description of the population

**N = 4189**	**Mean**	**SD**	**Range**
Age (years)	63	7	50–96
% men (%)	46.7		
Weight (Kg)	75	15	31–156
Height (cm)	167	9	102–197
BMI (Kg/m^2^)	27	5	13–60
Diabetes (%)	11.9%		
Hypertension (%)	47.2%		
Current smoking	18.2%		
Creatinine (mg/dL)	0.89	0.21	
Cystatin C (mg/L)	0.85	0.17	
MDRD (mL/min/1.73 m^2^)	78	17	
CKD-EPI (mL/min/1.73 m^2^)	81	15	
CKD-EPI Cys (mL/min/1.73 m^2^)	92	17	
CKD-EPI mix (mL/min/1.73 m^2^)	88	16	

Data are expressed as mean ± SD. Prevalence of CKD (defined as a GFR less than 60 mL/min/1.73 m^2^) is expressed in %.

**Table 2:** Prevalence (%) of CKD stages according to the equations used

	**MDRD**	**CKD-EPI**	**CKD-EPI Cys**	**CKD-EPI Mix**
All (n = 4189)	13	9.8	4.7	5
>90 mL/min/1.73 m^2^	22.1	33	63.2	48.9
60–89 mL/min/1.73 m^2^	64.8	57.2	32	46.1
30–59 mL/min/1.73 m^2^	12.9	9.6	4.5	4.8
<30 mL/min/1.73 m^2^	0.2	0.3	0.2	0.2

### Concordance analyses

Kappa statistics showed good agreement between the MDRD and CKD-EPI equations to detect stage 3 CKD (κ = 0.84). They also showed good agreement between the CKD-EPI Cys and the CKD-EPI mix equations (κ = 0.71). On the contrary, the kappa agreement range was only moderate or fair between the two creatinine-based equations and the two cystatin C-based equations (κ between 0.32 and 0.59). All kappa results between equations are summarized in Table 3.

**Table 3:** Kappa coefficients between the equations. 95% confidence intervals are shown between brackets

	**MDRD**	**CKD-EPI**	**CKD-EPI Cys**	**CKD-EPI mix**
MDRD	XXXXXXXXX	0.84 [0.81–0.86]	0.32 [028–0.36]	0.48 [0.44–0.53]
CKD-EPI		XXXXXXXXX	0.39 [0.33–0.44]	0.59 [0.55–0.64]
CKD-EPI Cys			XXXXXXXXX	0.71 [0.66–0.76]
CKD-EPI mix				XXXXXXXXX

**Figure 1:**
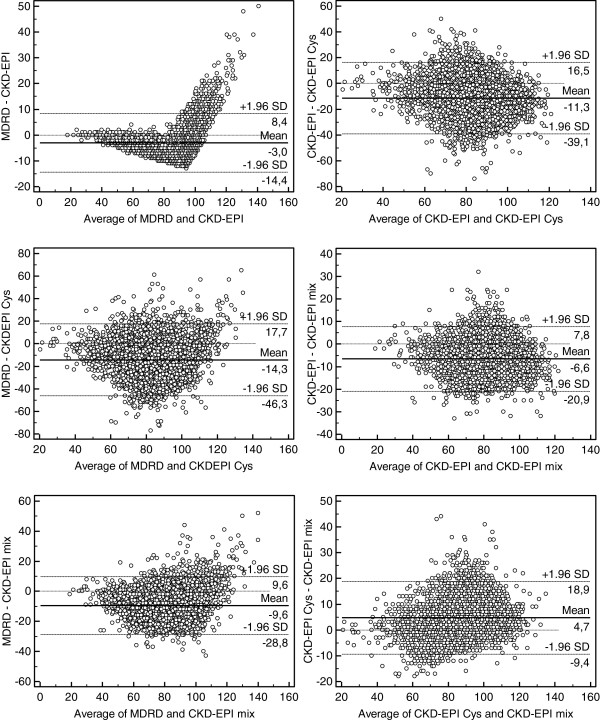
**Bland and Altman analysis between the MDRD study, the CKD-EPI, the CKD-EPI Cys and the CKD-EPI mix equations.** The continuous line represents the mean difference between measured and estimated GFR, whereas the dashed lines represent the limits of agreement (mean difference ± 2 SD). All values are expressed in mL/min/1.73 m^2^.

The Bland and Altman analyses are given in Figure 1. Once again, because no equation can be considered as the perfect reference, we only considered the absolute bias. In Table 4, the bias (mean difference) and the SD around the bias are summarized. The larger differences are observed between the creatinine- and the cystatin C-based equations although differences between the MDRD and the CKD-EPI and between the CKD-EPI Cys and CKD-EPI mix are lower.

**Table 4:** Absolute bias (mean difference) (bold) and precision (SD around the bias) (italic) between the equations

	**MDRD**	**CKD-EPI**	**CKD-EPI Cys**	**CKD-EPI mix**
MDRD	XXXXXXXXX	**5.8**	**16.3**	**9.8**
CKD-EPI	*3*	XXXXXXXXX	**14.2**	**7.3**
CKD-EPI Cys	*14.3*	*11.3*	XXXXXXXXX	**7.2**
CKD-EPI mix	*9.6*	*6.6*	*4.7*	XXXXXXXXX

All data are expressed in mL/min/1.73 m^2^.

### Subgroups analyses according to gender, diabetic status and age

No difference was observed between stage 3 CKD prevalence according to gender when either CKD-EPI Cys or CKD-EPI mix were used (Additional file 1: Table S2). On the contrary, the prevalence of stage 3 CKD was significantly higher in women when the CKD-EPI equation was used and even more when the MDRD study equation was considered.

We also calculated the prevalence of stage 3 CKD in diabetic patients. In these patients, the prevalence with the MDRD, CKD-EPI, CKD-EPI Cys and CKD-EPI mix were 17.1%, 14.5%, 10.2% and 10.4%, respectively.

**Figure 2:**
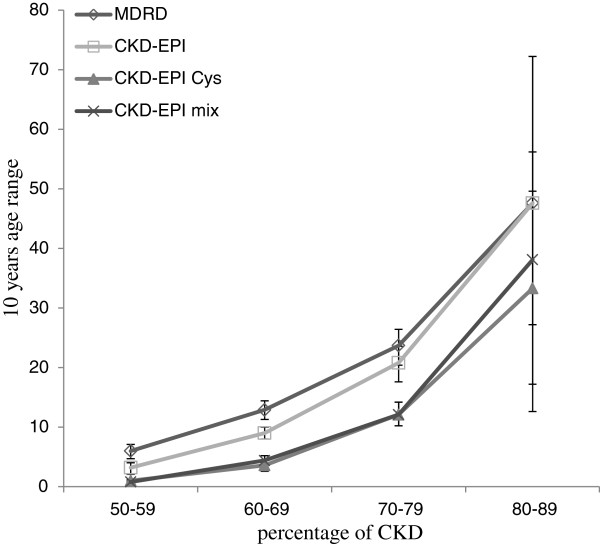
Prevalence (in % with the 95% confidence interval) of CKD according to age (X axis: 10 years age range) (Y axis: percentage).

In Table 5 and Figure 2, we summarized the prevalence of stage 3 CKD according to age. As expected, the prevalence of CKD increased with age, regardless of the equation used. The prevalence of CKD showed a significant difference between the two creatinine-based equations and the two cystatin C-based equations for all age ranges, except for those older than 80 years. Whatever the age range, we observed no difference between the CKD-EPI Cys and the CKD-EPI mix equations. On the contrary, difference of prevalence between the MDRD and CKD-EPI equations was not significant in those older than 70 years.

**Table 5:** Prevalence of CKD according to age (age ranges of 10 years) (in %)

	**Whole (n = 4208)**	**50-59 (n = 1455)**	**60-69 (n = 1812)**	**70-79 (n = 919)**	**80-89 (n = 21)**
MDRD	13	6	12.9	23.7	47.6
CKD-EPI	9.8 *	3.2 *	9 *	20.8	47.6
CKD-EPI Cys	4.7 *^$^	1 *^$^	3.6 *^$^	12.1 *^$^	33.3
CKD-EPI mix	5 *^$^	0.8 *^$^	4.4 *^$^	12.1 *^$^	38.1

*p < 0.05 compared with the MDRD, ^$^ p < 0.05 compared with the CKD-EPI, ^§^ p < 0.05 compared with the CKD-EPI Cys.

### Analysis of discrepant results

In Table 6, we showed the percentage of patients with discrepant results (eGFR > or < 60 mL/min/1.73 m^2^) according to the equation used. Discrepant results (3.3%, n = 140) were observed between the MDRD and the CKD-EPI equations. In these subjects, the MDRD-based results were systematically indicating CKD whereas the CKD-EPI equation results were greater than 60 mL/min/1.73 m^2^. Comparable percentage of discrepancy (2.7%, n = 114) was observed between the CKD-EPI Cys and the CKD-EPI mix but with these equations, the differences between positive and negative screening were not uniform. Indeed, in 45% of cases, the CKD-EPI Cys indicated stage 3 CKD whereas the CKD-EPI mix result was greater than 60 mL/min/1.73 m^2^ and the opposite was observed in 55% of cases. The highest percentage of discrepant results was observed between the MDRD and the CKD-EPI Cys equations (11.3%, n = 473). An 8% discrepancy was observed between the CKD-EPI and the CKD-EPI Cys and between the MDRD and the CKD-EPI mix. The percentage of discrepancies between CKD-EPI and CKD-EPI mix was intermediate at 5.5%. Regarding these last comparisons, creatinine-based equations gave positive result although cystatin-C based equations were reassuring for stage 3 CKD diagnosis in a majority of patients.

**Table 6:** Percentage of discordant patients according to the two equations considered

	**MDRD**	**CKD-EPI**	**CKD-EPI Cys**	**CKD-EPI mix**
MDRD	XXXXXXXXX			
CKD-EPI	3.3% (99.98%)	XXXXXXXXX		
CKD-EPI Cys	11.2% (87.1%)	8.29% (81.5%)	XXXXXXXXX	
CKD-EPI mix	8.63% (96.7%)	5.58% (92.8%)	2.71% (44.7%)	XXXXXXXXX

In the brackets is the percentage of patients, among these discordant patients, yielding a positive result (eGFR below 60 mL/min/1.73 m^2^) when the equation in the column is considered (although the equation in the same line gives an eGFR greater than 60 mL/min/1.73 m^2^).

Table 7 summarized the possible combination of screening results. Some combinations were not or very rarely (n < 10) found. For example, a positive screening with the two cystatin C-based equations, but not with the creatinine-based equations was found only in 11 subjects. The four equations yielded the same results (positive or negative screening) in 3702 cases (89%). In 131 subjects, only the MDRD equation gave a positive screening result, although the three other equations indicated an eGFR greater than 60 mL/min/1.73 m^2^. Compared to the whole population, these patients were more often women (23.7% versus 46.8%, respectively) and were lighter and shorter. In 218 subjects, both MDRD and CKD-EPI equations gave a positive screening result although the results were negative for CKD when using the CKD-EPI Cys and CKD-EPI mix equations. The mean age of these discrepant subjects was significantly higher compared to the whole population (67 ± 7 versus 63 ± 7 years old, respectively). Similarly, patients with positive screening for all equations except CKD-EPI Cys (n = 63) were older (68 ± 5 versus 63 ± 7 years old, respectively) and more frequently women (28.6% versus 46.8%, respectively). Inversely, patients with a positive screening only for CKD-EPI Cys (n = 49) were older (68 ± 7 versus 63 ± 7 years old, respectively) and heavier (BMI at 30 ± 6 versus 27 ± 5 kg/m^2^, respectively).

**Table 7:** Characteristics of the subjects in the different subgroups

	**Whole**	**MDRD>60**	**MDRD<60**	**MDRD<60**	**MDRD<60**	**MDRD<60**	**MDRD>60**
		**CKD-EPI>60**	**CKD-EPI<60**	**CKD-EPI>60**	**CKD-EPI<60**	**CKD-EPI<60**	**CKD-EPI>60**
		**CKD-EPI Cys>60**	**CKD-EPI Cys<60**	**CKD-EPI Cys>60**	**CKD-EPI Cys>60**	**CKD-EPI Cys>60**	**CKD-EPI Cys<60**
		**CKD-EPI mix>60**	**CKD-EPI mix<60**	**CKD-EPI mix>60**	**CKD-EPI mix>60**	**CKD-EPI mix<60**	**CKD-EPI mix>60**
N	4189	3573	129	131	218	63	49
Age (years)	63 ± 7	62 ± 7*	71 ± 6*	63 ± 7	67 ± 7*	68 ± 5*	68 ± 7 *
Men (%)	46.7	48.2	48.1	23.7 *	40.8	28.6 *	46.9
Weight (Kg)	75 ± 15	75 ± 15	80 ± 17*	72 ± 14*	75 ± 14	77 ± 12	82 ± 20*
Height (cm)	167 ± 9	167 ± 9	166 ± 9	164 ± 9*	166 ± 9	165 ± 8	166 ± 9
BMI (Kg/m^2^)	27 ± 5	27 ± 5	29 ± 5*	27 ± 4	27 ± 4	28 ± 4	30 ± 6 *
Diabetes (%)	11.9	11.1	25.6*	8.4	11	22.2*	24.5*
HTA (%)	47.2	45.1	72.9*	50.4	51.4	63.5*	75.5*

HTA is hypertension. *p < 0.05 compared to the whole population.

## Discussion

Epidemiologic studies in Western countries have shown that prevalence of stage 3 CKD, defined as a GFR less than 60 mL/min/1.73 m^2^, is about 10% in the general population [1,2,11,27]. But there are large differences between studies regarding the prevalence of CKD which can be explained not only by the population characteristics, but also by the difference in the method used to estimate GFR [2]. Most of the recent data have been obtained with the MDRD study equation using a well calibrated serum creatinine [2,28,29]. However, the use of this equation is not free from criticisms. We and others, have demonstrated that this equation tends to strongly underestimate GFR in healthy populations and, more generally, in patients with normal or near normal creatinine values [12,30,31]. Thus, the Levey’s group has recently proposed a new equation, the CKD-EPI equation, which is expected to be better especially in the higher GFR range (over 60 ml/min/1.73 m^2^). Indeed, the CKD prevalence is significantly lower according to the CKD-EPI equations for the present population cohort than if the MDRD equation is used (9.8% versus 13%). Among the patients screened, 140 (3.3%) were classified as having stage 3 CKD with the MDRD study equation but not with the CKD-EPI equation. This is important from an epidemiological point of view. We have thus confirmed that a higher prevalence of stage 3 CKD when the MDRD equation is used [11,32-36]. The higher prevalence of stage 3 CKD found with the MDRD study equation compared to the prevalence observed with the newer CKD-EPI equation may be explained by the systematic underestimation of GFR obtained with this first equation, especially in women [12,30,35,37]. Interestingly, we confirmed that difference between the MDRD and the CKD-EPI equations seems to decrease with age, notably in subjects older than 70 years [34-38]. These results may be explained by the performances of the two equations which have been showed to be very similar (or at least less different) in elderly patients [39]. Indeed, it has been shown that the MDRD equation yields systematically lower eGFR results (for a given age and creatinine level) than the CKD-EPI equation except in the very old patient [35]. This is confirmed by our own data showing that all patients presenting discrepant results between MDRD and CKD-EPI equations are actually positively screened only by the MDRD equation, except in one patient who was the oldest of the sample (96 years old).

In their initial CKD-EPI article [11], Levey et al. have also compared prevalence of CKD in the NHANES study. If we consider the same definition of CKD (only based on eGFR), these authors have found that prevalence of CKD in the NHANES study was 10.06% with the CKD-EPI equation and 14.9% with the MDRD study equation (Appendix Table 9 in [11]) in subjects aged between 60 and 69 years. The discrepancies observed between the two equations are comparable to discrepancies observed in our study.

One can conclude that the performance of the CKD-EPI equation to determine the prevalence of CKD in epidemiological studies is better than the MDRD study equation [11,32]. However, this equation is not free from criticism. The CKD-EPI notably remains dependent on the limitations of serum creatinine and its precision is imperfect. Cystatin C is often presented as a better marker of kidney function, especially because its concentration is less influenced by muscular mass than serum creatinine [15]. New equations based on standardized cystatin C have been recently proposed by the CKD-EPI consortium. These equations have been built and internally validated from an impressive sample (5592 subjects in the developing dataset and 1119 in the validation dataset). The authors demonstrated better performance of the equations combining creatinine and cystatin C compared to the CKD-EPI equation [23]. Recent studies confirmed the good performances of the two cystatin C-based equations in specific populations [24,40]. This better performance is also supported by the studies that show a better estimate of mortality risk when using cystatin C compared to creatinine-based equations [41,42].

In our population of volunteers, the prevalence of stage 3 CKD is strongly discordant using creatinine- or cystatin C-based equations. Indeed, if the prevalence of CKD is as high as 13% and 9.8% using the MDRD or the CKD-EPI study equations, the prevalence will decrease to 5%, for the equations based on cystatin C only or on cystatin C and creatinine. Interestingly, the seminal study on the cystatin C-based equations published by Inker et al. showed a better performance of the CKD-EPI mix equation to estimate GFR [23]. In our study, we found however no difference in the prevalence of stage 3 CKD results between the CKD-EPI Cys or the CKD-EPI mix. This may be related to the way the authors calibrated the cystatin C measurement, which was not unquestionable. Also, the bias of the equations influences the epidemiologic results and we note that in the CKD-EPI study (Table 3 in [43]), bias of the CKD-EPI Cys is similar to the bias of the CKD-EPI mix equation in the range of interest for the CKD screening (i.e. around 60 mL/min/1.73 m^2^).

In our cohort, when we considered the ability of each equation to detect CKD, kappa statistics showed very good agreement (κ = 0.84) between the CKD-EPI and the MDRD equations. Agreement is still acceptable, even if lower (κ = 0.71), between the CKD-EPI Cys and the CKD-EPI mix. However, agreement between CKD-EPI (or MDRD) and CKD-EPI Cys is low (0.39 and 0.32, respectively) although agreement is slightly better between CKD-EPI (and MDRD) and the CKD-EPI mix equations (0.59 and 0.48, respectively). These results underlined the powerful value of each biomarker in the equations: more concordant results between equations based exclusively on creatinine (MDRD versus CKD-EPI), poor concordant results between equations based on creatinine and on cystatin C only (CKD-EPI or MDRD versus CKD-EPI Cys), and intermediate concordance results when the mix equation is compared to others. Better concordance between the CKD-EPI and the two new cystatin C-based equations compared to the MDRD study equation is also probably explained by the mathematical construction of the equation (different exponent applied to creatinine according to creatinine level) and by the fact that the MDRD study equation has been developed on a very different sample [44] although the three other equations have been developed from comparable cohorts [11,23].

Bias and precision results must be interpreted with caution in our study. Indeed, the systematic difference between the MDRD and the CKD-EPI equations seems low (3 mL/min/1.73 m^2^). It can be suggested from Figure 1 that this bias is however very dependent on the GFR level. The systematic difference between the MDRD and the CKD-EPI equations increases with increasing GFR values especially at high values. Comparing the three CKD-EPI equations, we also found a positive correlation between the difference in equations and the mean of the two equations, which means that the differences between equations slightly increase with eGFR levels. This correlation is however low (r^2^ between 0.02 and 0.04, p < 0.001) and linear although a clear non linear relationship with a knot join-point around X = 80 mL/min/1.73 m^2^ does exist when MDRD is compared to CKD-EPI and CKD-EPI Mix. This fact also means that discrepancies between the MDRD and the CKD-EPI are systematic and almost in the same direction, i.e. MDRD giving a positive screening result and CKD-EPI giving a negative one. Such a systematic deduction cannot be done when discrepancies are found between the CKD-EPI Cys and the CKD-EPI mix equations.

The prevalence of stage 3 CKD may be higher in women if the CKD-EPI, and especially if the MDRD study equation is used [2,11]. Contrary to the CKD-EPI and the MDRD study equations, the prevalence of stage 3 CKD with the cystatin C-based equations is the same in both men and women. In the absence of mGFR, this discrepancy in results according to gender cannot be explained. Regarding the underestimation of women’s GFR by the MDRD equation [30], the higher prevalence of stage 3 CKD in women must be interpreted with caution. Differences in prevalence of CKD according to gender observed between the three CKD-EPI equations deserve further studies.

Diabetes is a well-known risk factor associated with CKD. As expected, CKD prevalence was higher in diabetic patients than in non-diabetic patients. Interestingly, the difference between the prevalences with creatinine-based versus the cystatin C-based equations appears less important than in the general population. Once again, in the absence of measured GFR, we can only describe such differences. Moreover, we must be careful in our interpretation of this result, as diabetic patients have also different clinical characteristics than non-diabetics, like a higher mean BMI.

CKD prevalence is also greatly influenced by age. As expected, we observed an increasing prevalence of stage 3 CKD according to age [2,23,37,45,46]. At every age range, the prevalence of stage 3 CKD remains significantly lower with the CKD-EPI Cys and the CKD-EPI mix, except in the higher age category but results must be interpreted with caution due to the low sample number.

It is useful to analyze the characteristics of patients with discrepant results. Generally, concordant results for the 4 equations were found in 3702 subjects (88%). If we based our CKD definition on eGFR less than 60 mL/min/1.73 m^2^ with all four equations, the prevalence of stage 3 CKD would be 3.1%. Our first comment must be emphasized: a perfect concordance between all four equations does not necessarily mean that the results of these equations are correct. Indeed, in the absence of mGFR, it remains possible that all four equations misclassified the patients. The same argument must be applied in the following discussion on discrepant subjects. In 131 subjects, the discrepant result is the positive screening with the MDRD study equation. As already discussed, there is little doubt that the MDRD effectively underestimates GFR and thus yields a substantial proportion of false positive screening results. The most numerous discrepant patients are those with a positive screening result with the creatinine-based equations (MDRD and CKD-EPI) but with a negative screening result with the two cystatin C-based equations. These 218 patients are slightly older compared to the general population (67 ± 7 y versus 63 ± 7 y). Because mGFR is not really known, such a discrepancy remains difficult to explain. However, this last result could be interpreted in the light of recent studies suggesting that the combined creatinine and cystatin-C based equations better estimate GFR in the elderly subject [24,45]. Moreover, it must be underlined that the proportion of diabetic and hypertensive subjects was the same in the whole population. The positive screening result using cystatin C-based equations could thus be the most accurate, especially in the elderly [46]. In 63 patients, the CKD-EPI Cys was the only one to give a negative screening result. These subjects were slightly older and more often women (71.4%). On the contrary, the CKD-EPI Cys is the only equation to give a positive screening result in 49 subjects. These subjects were older and had a higher BMI. Once again, in the absence of mGFR, we can only note the discrepancies without asserting which equation actually gives the better results. The possible influence of body mass index on cystatin C levels deserves future study [47]. Further studies are actually important in these discrepant patients who are also more frequently hypertensive and diabetic.

There are several limitations to our study. First, the main limitation is linked to the fact that we have not measured GFR with a reference method. Therefore, even if we have indirect arguments to affirm that the CKD-EPI and MDRD equations overestimate the prevalence of CKD, such an assertion is conclusive only if a reference method to measure GFR is used. In our cohort, we can only identify clinical characteristics of discrepant patients. In the same vein, our Jaffe assay has lesser precision than enzymatic assays, although IDMS traceable*.* Second, our population may not be representative of the Belgian population because only volunteers were included. We actually noted a higher proportion of hypertension and diabetes than expected in the general population. Therefore, our results are not generally applicable for epidemiological considerations but more of an illustration of potential discrepancies in the CKD epidemiology due to difference in estimating GFR [21,32]. Third, we have no data on the ethnicity. As the ethnicity factor for each equation is different, this could be source of bias. But in the Province of Liège, Caucasians are by far the dominant ethnic group. Therefore, it is doubtful that the differences observed in our study are due to ethnic factors. Fourth, as in several epidemiological studies, our subjects have been tested only one time. A strict definition of CKD requires that two or three samples confirm the first result after at least three months [9]. With this correct definition, CKD prevalence would likely still be lower [48-52]. Last, we have defined stage 3 CKD as a GFR less than 60 mL/min/1.73 m^2^. The definition of CKD is however subject to debate and we have recently questioned this definition, notably in elderly population [31,37,46].

## Conclusions

The present study has illustrated large discrepancies for the prevalence of stage 3 CKD according to the biomarker used to estimate the GFR. Moving from strictly creatinine-based equations (MDRD or CKD-EPI) to cystatin C-based or combined equations will decrease prevalence of stage 3 CKD by half, which is highly significant from an epidemiological point of view. Additional studies are thus necessary before asserting we know the true prevalence of CKD in the general population.

## Abbreviations

BMI: Body mass index; CKD: Chronic kidney disease; CKD-EPI: Chronic Kidney disease epidemiology collaboration study; CKD-EPI Cys: CKD-EPI equation based on cystatin C alone; CKD-EPI mix: CKD-EPI equation based on creatinine and cystatin C; eGFR: Estimated glomerular filtration rate; GFR: Glomerular filtration rate; MDRD: Modified diet in renal disease; mGFR: Measured glomerular filtration rate; NHANES: National health and nutrition examination survey; PENIA: Particle-enhanced nephelometric immunoassay.

## Competing interests

The authors declare that they have no competing interests.

## Authors’ contributions

PD is the principal investigator and the first author of this manuscript. EC is the Biochemist who measured serum creatinine. LL measured cystatin C. OM and OB performed statistical analyses. J-MK, EC, OM and OB have critically corrected the manuscript as Chief of the Department of Nephrology, Clinical Chemistry and Public Health, Epidemiology and Health Economics, respectively. All authors read and approved the final manuscript.

## Pre-publication history

The pre-publication history for this paper can be accessed here:

http://www.biomedcentral.com/1471-2369/14/57/prepub

## Supplementary Material

Additional files 1: Table S1 Creatinine and cystatin C-based equations for glomerular filtration rate (GFR) estimation. Serum creatinine: SCr in mg/dL, serum cystatin C: SCys in mg/L. **Table S2.** Prevalence of CKD according to gender (in %). Click here for file
