# Supplementary material for: Creatinine-or cystatin C-based equations to estimate glomerular filtration in the general population: impact on the epidemiology of chronic kidney disease
Source: BMC Nephrol. 2013 Mar 12;14:57. doi: 10.1186/1471-2369-14-57 (PMC3637126; doi:10.1186/1471-2369-14-57)
Supplement: Additional files 1: Table S1 — Creatinine and cystatin C-based equations for glomerular filtration rate (GFR) estimation. Serum creatinine: SCr in mg/dL, serum cystatin C: SCys in mg/L. Table S2. Prevalence of CKD according to gender (in %). [file 1471-2369-14-57-S1.docx]

**Supplementary files**

**Table S1:** Creatinine and cystatin C-based equations for glomerular filtration rate (GFR) estimation. Serum creatinine: SCr in mg/dL, serum cystatine C : SCys in mg/L.

| MDRD |  | 175 x Scr^-1.154^ x age^-0.203^ x [0.742 if female] |
| --- | --- | --- |
| CKD-EPI |  |  |
| Female | SCr ≤ 0.7 mg/dL | 144 x (Scr/0.7)^-0.329^ x 0.993^age^ |
|  | SCr > 0.7 mg/dL | 144 x (Scr/0.7)^-1.209^ x 0.993^age^ |
| Male | SCr ≤ 0.9 mg/dL | 141x (Scr/0.9)^-0.411^ x 0.993^age^ |
|  | SCr > 0.9 mg/dL | 141x (Scr/0.9)^-1.209^ x 0.993^age^ |
| CKD-EPI Cyst | SCyst ≤ 0.8 mg/L | 133 x (Scyst/0,8)^-0.499^ x 0.996^age^ [x0.932 if female] |
|  | SCyst >0.8 mg/L | 133 x (Scys/0,8)^-1.328^ x 0.996^age^ [x0.932 if female] |
| CKD-EPI Mix |  |  |
| Female | SCr ≤ 0.7 mg/dL and SCyst ≤ 0.8 mg/dL | 130 x (Scr/0.7)^-0.248^ x (Scyst/0.8)^-0.375^ x 0.995^age^ |
|  | SCr ≤ 0.7 mg/dL and SCyst > 0.8 mg/dL | 130 x (Scr/0.7)^-0.248^ x (Scyst/0.8)^-0.711^ x 0.995^age^ |
|  | SCr>0.7 mg/dL and SCyst ≤ 0.8 mg/dL | 130 x (Scr/0.7)^-0.601^ x (Scyst/0.8)^-0.375^ x 0.995^age^ |
|  | SCr >0.7 mg/dL and SCyst ≥ 0.8 mg/dL | 130 x (Scr/0.7)^-0.601^ x (Scyst/0.8)^-0.711^ x 0.995^age^ |
| Male | SCr ≤0.9 mg/dL and SCyst ≤ 0.8 mg/dL | 135 x (Scr/0.9)^-0.207^ x (Scyst/0.8)^-0.375^ x 0.995^age^ |
|  | SCr≤0.9 mg/dL and SCyst > 0.8 mg/dL | 135 x (Scr/0.9)^-0.207^ x (Scyst/0.8)^-0.711^ x 0.995^age^ |
|  | SCr >0.9 mg/dL and SCyst ≤ 0.8 mg/dL | 135 x (Scr/0.9)^-0.601^ x (Scyst/0.8)^-0.375^ x 0.995^age^ |
|  | SCr >0.9 mg/dL and SCyst > 0.8 mg/dL | 135 x (Scr/0.7)^-0.601^ x (Scyst/0.8)^-0.711^ x 0.995^age^ |

**Table S2:** Prevalence of CKD according to gender (in %).

| **eGFR** | **Men (n=1969)** | **Women (n=2239)** | **Difference** |
| --- | --- | --- | --- |
| MDRD | 10% | 15% | P<0.0001 |
| CKD-EPI | 9% | 11% | P=0.036 |
| CKD-EPI Cys | 5% | 5% | NS |
| CKD-EPI mix | 5% | 5% | NS |
